# Investigation on Eigenfrequency of a Cylindrical Shell Resonator under Resonator-Top Trimming Methods

**DOI:** 10.3390/s17092011

**Published:** 2017-09-02

**Authors:** Kai Zeng, Youwang Hu, Guiling Deng, Xiaoyan Sun, Wenyi Su, Yunpeng Lu, Ji’an Duan

**Affiliations:** State Key Laboratory of High Performance and Complex Manufacturing, College of Mechanical and Electrical Engineering, Central South University, Changsha 410083, China; zengkai@csu.edu.cn (K.Z.); gldeng@csu.edu.cn (G.D.); sunxy@csu.edu.cn (X.S.); steven_su@csu.edu.cn (W.S.); luyunpeng@csu.edu.cn (Y.L.); duanjian@csu.edu.cn (J.D.)

**Keywords:** eigenfrequency, cylindrical resonator, trimming methods, femtosecond laser

## Abstract

The eigenfrequency of a resonator plays a significant role in the operation of a cylindrical shell vibrating gyroscope, and trimming is aimed at eliminating the frequency split that is the difference of eigenfrequency between two work modes. In this paper, the effects on eigenfrequency under resonator-top trimming methods that trim the top of the resonator wall are investigated by simulation and experiments. Simulation results show that the eigenfrequency of the trimmed mode increases in the holes-trimming method, whereas it decreases in the grooves-trimming method. At the same time, the untrimmed modes decrease in both holes-trimming and grooves-trimming methods. Moreover, grooves-trimming is more efficient than holes-trimming, which indicates that grooves-trimming can be a primary trimming method, and holes-trimming can be a precision trimming method. The rigidity condition after grooves-trimming is also studied to explain the variation of eigenfrequency. A femtosecond laser is employed in the resonator trimming experiment by the precise ablation of the material. Experimental results are in agreement with the simulation results.

## 1. Introduction

A cylindrical shell vibrating gyroscope is a kind of solid-state wave gyroscope which senses angular velocity based on the inertial effect of the standing wave in a cylindrical shell resonator, and it has the advantages of a simple structure, high operational accuracy, low cost, and long life [1,2]. Therefore, these advantages render the cylindrical gyroscope useful in numerous applications such as avionics systems, borehole surveying, missiles, naval equipment, platform stabilization, and robotics [3]. However, in order to achieve these advantages, a precise resonator, which is susceptible to unavoidable fabrication errors, is necessary [4]. Hence, the resonator trimming after fabrication is of great importance to improving the performance of gyroscopes.

More specifically, fabrication errors always destroy the symmetry of resonator structure, and lead to the splitting of eigenfrequencies and the offsetting of eigenmodes, which is the reason for quadrature error in the gyroscope [5]. Meanwhile, the offset of eigenmodes is significantly affected by frequency split [6]; therefore, eigenfrequency becomes the key factor in improving the performance of gyroscopes. Electrical and mechanical balancing are two common trimming methods used to eliminate the frequency split. Electrical balancing methods can only regulate the eigenfrequency on a small scale [7,8], whereas mechanical balancing can eliminate large frequency split. Conventionally, mechanical balance, such as drilling holes on the button and grooving on the top of the resonator [9], changes local stiffness and mass distribution remarkably to rapidly reduce frequency split. In addition, frequency split could be eliminated by removing masses at pre-selected locations around the resonator, and the magnitude of the trimming masses can be calculated easily [10,11]. Another kind of trimming principle aimed at the variation of mass and rigidity has been presented [12], and trimming holes and grooves on the resonator have been shown to be effective, precise, and viable methods. Under these trimming methods, stress effects under varying temperature [13], Q factor [14], and node position [15] are studied. However, studies on the effects on eigenfrequency after trimming are rare; therefore, this paper focuses on eigenfrequency. On the other hand, the trimming tool employed is also important in the trimming process. For a thin cylindrical wall, mechanical drilling and milling causes large deformation, residual stress, and high cost, and previous methods that trim holes on the bottom plate [2,6,9] have aimed at reducing these disadvantages. Laser ablation does not deform the resonator, but causes thermal stress, whereas femtosecond laser results in a lower thermal stress [16,17]. The advantages of femtosecond lasers for precise material processing are discussed and demonstrated in References [18,19], which guarantee the trimming of a tiny material. Hence, femtosecond laser is employed in our experiment to reach a high processing accuracy.

In this paper, the effects on eigenfrequency under holes-trimming and grooves-trimming methods for a cylindrical shell vibrating gyroscope are investigated by simulation at first. Holes-trimming is processed on the top of the resonator wall, which is benefitted from femtosecond laser. Grooves-trimming is processed on the top edge of the resonator, which has fewer side-effects on the resonator. In addition, the rigidity condition after rigidity trimming is researched to explain the changes of eigenfrequency. Experiments are also conducted in this work to validate the simulation results.

## 2. Structure and Working Principle

**Figure 1:**
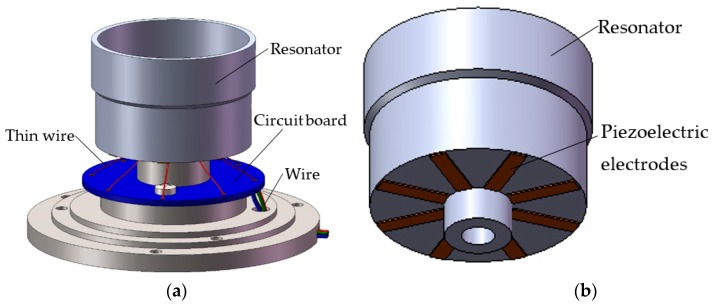
Configuration of cylindrical shell gyroscope. (**a**) Gyroscope; (**b**) Resonator.

As shown in Figure 1, the structure of a typical cylindrical shell vibratory gyroscope comprises a cylindrical resonator, a circuit board, and eight piezoelectric electrodes that are glued on the bottom of the resonator. The vibration of the gyroscope is excited and then converted into a single voltage by the use of piezoelectric strips sandwiched between the electrodes. A thin wire connects the circuit board with the electrodes, and another wire connects the gyroscope with the control device.

**Figure 2:**
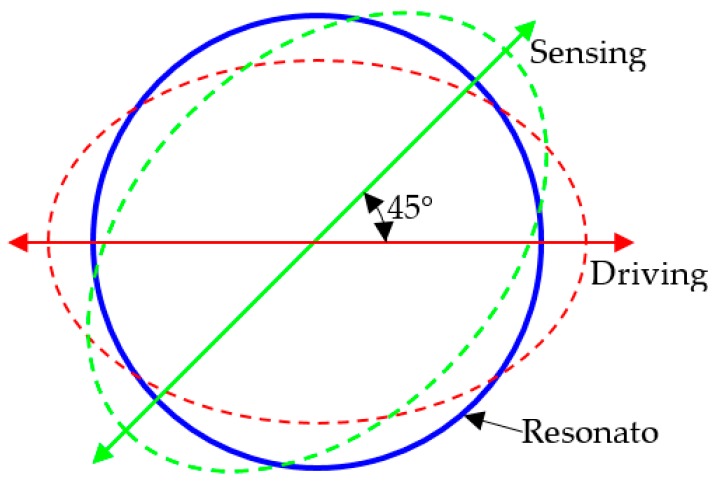
Mode shape of the resonator.

The schematic diagram of the working principle is shown in Figure 2. When an alternating voltage is applied to the piezoelectric electrodes that coincide with the driving mode, for the converse-piezoelectric effect, the piezoelectric electrodes contract and expand, which produce an alternating bending moment on the resonator, hence the resonator is induced into a circle-ellipse flexural vibration in the driving mode at the frequency of the alternating voltage. To achieve the maximum amplitude of vibration, the frequency of the excited voltage should be consistent with the eigenfrequency of the driving mode, which is easily achievable by controlling the power supply.

While the resonator vibrates in the driving mode, and the gyroscope is rotating about its axis, the Coriolis force in the resonator, which is caused by the Coriolis Effect, would excite the resonator into another circle-ellipse flexural vibration in the sensing mode at the same frequency of the driving mode. For the same reason, the resonator should vibrate in the eigenfrequency of the sensing mode, which requires trimming the eigenfrequencies of the driving and sensing modes to be equal. The resonator vibrates in the sensing mode, which is separated by 45 degrees from the driving mode, and vibrations are converted into an electric signal by the use of piezoelectric strips sandwiched between the electrodes. The electric signal is proportional to angular velocity, and can be transferred to external devices via a wire.

## 3. Simulation

In order to obtain the numerical solutions in the study, FEM software ANSYS is utilized to analyze the eigenfrequency of the resonator under different trimming methods. Because the mesh method and size are of great importance to the simulation accuracy, the resonator is completely meshed with hexahedron elements with a size of 0.2 mm, which only induces a frequency split of 5 × 10 ^−6^ Hz to a perfect resonator (0.01 Hz is quite small in practice). The formula is as follows:

(1) $$\omega =\sqrt{\frac{K^{*}}{m^{*}}},$$

where eigenfrequency ω is directly related to the equivalent rigidity k* and equivalent mass m* of the resonator. Therefore, the effects can be explained briefly by analyzing the changes of equivalent rigidity and equivalent mass. Two kinds of resonator-top trimming methods including holes-trimming and grooves-trimming are exerted on the finite element model in order to reveal their effect on the eigenfrequency. By means of modal analysis, the eigenfrequencies of both the driving and sensing modes are extracted. The difference of eigenfrequency between the driving mode and sensing mode is considered to be the frequency split.

### 3.1. Finite Element Modeling

**Figure 3:**
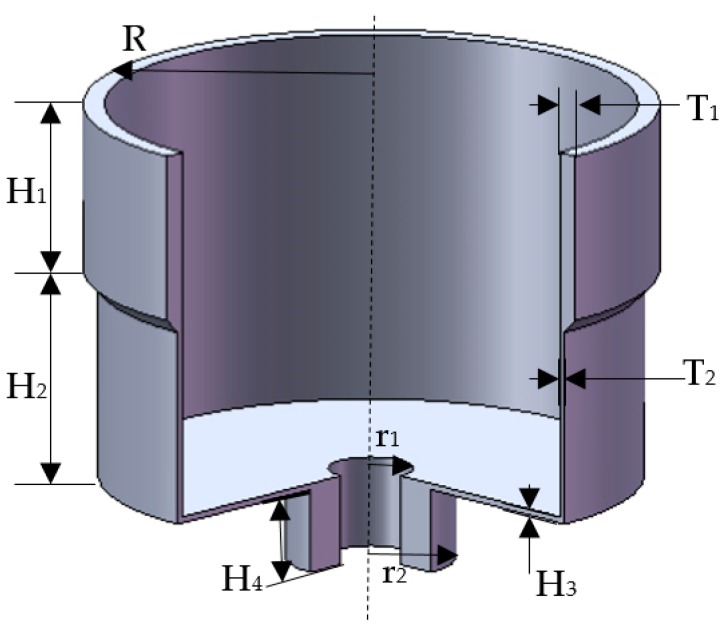
The resonator model.

The material properties listed in Table 1, and the geometry parameters listed in Table 2 are used to build the model (shown in Figure 3). In addition, the element type used to mesh the resonator is SOLID 186.

### 3.2. Effect of Holes-Trimming on Eigenfrequency

**Figure 4:**
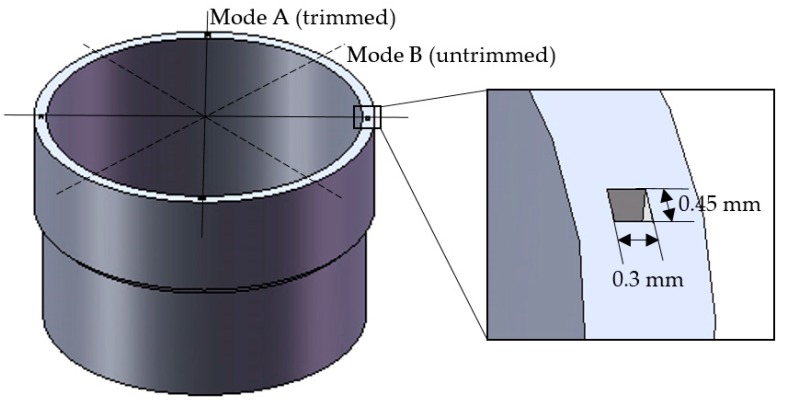
Drilling holes on the top of the resonator.

As shown in Figure 4, four 0.3 mm × 0.45 mm rectangular holes were formed on the top of the resonator wall to change its eigenfrequency. In order to guarantee static balance, four symmetrical holes that were distributed 90° from each other are trimmed. This is an excellent way to maintain the continuity of the resonator structure, which would otherwise lead to a large decrease in rigidity. In practice, however, round holes are more common than rectangular hole. The reason for drilling a rectangle hole is to increase the accuracy of the finite element model, which has been validated by comparing the two kinds of holes.

**Figure 5:**
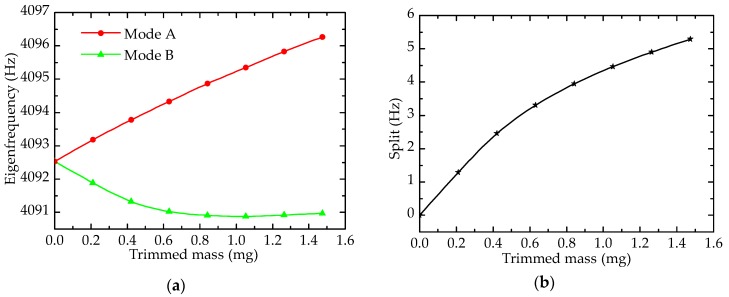
Effect of drilling holes on eigenfrequency: (**a**) Eigenfrequency; (**b**) Frequency split.

As shown in Figure 5a, at the beginning of trimming, the depth of hole is zero, the eigenfrequencies of mode A and mode B are almost equivalent (perfect resonator). Holes affect mass and rigidity simultaneously, and the decrease in mass would increase eigenfrequency, whereas the decrease in rigidity would reduce eigenfrequency. Changes of mass play a primary role in Mode A, and rigidity affects more in Mode B. Hence, with the increasing of trimmed mass (depth), the eigenfrequency of mode A increases almost linearly, and that of mode B decreases slowly. The frequency split, which is show in Figure 5b, is nearly zero before trimming, and it increases along with the addition of trimmed mass. It is worth mentioning that mode A (mode B) can be the driving mode or sensing mode, depending on the device connected with corresponding electrodes. Once the electrodes that are of the orientation of mode A are connected with power supply, the driving mode is located at mode A. The same is true for mode B.

### 3.3. Effect of Grooves-Trimming on Eigenfrequency

The continuity of the resonator structure is the key factor of rigidity, and forming grooves is a process to change continuity. A previous method involves forming grooves on the top of the resonator [9], which decreases its rigidity greatly (a decrease of 5% on the trimmed mode) and affects the Q factor heavily [14]. Hence, a novel grooves-trimming method that forms grooves on the top edge of the resonator is presented, which changes the rigidity only slightly (a decrease of 2% on the trimmed mode), and its effect on eigenfrequency is discussed as below.

#### 3.3.1. Effect of Forming Grooves on Eigenfrequency

**Figure 6:**
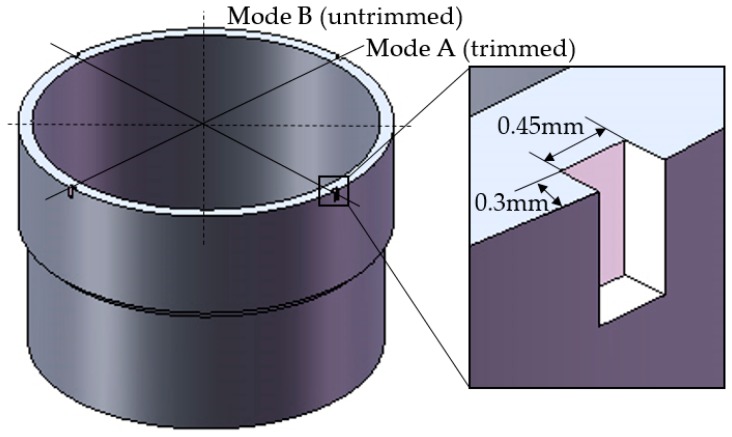
Forming grooves on the top edge of the resonator.

Grooves-trimming is shown in Figure 6; four 0.3 mm × 0.45 mm grooves are formed on the top edge of the resonator wall.

**Figure 7:**
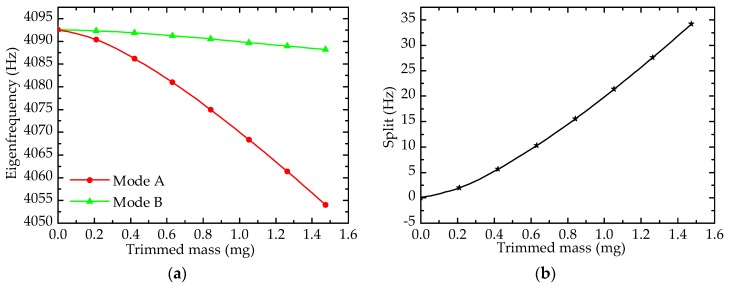
Effect of forming grooves on eigenfrequency: (**a**) Eigenfrequency; (**b**) Frequency split.

As shown in Figure 7, both the eigenfrequencies of mode A and mode B fall, but the eigenfrequency of mode B changes slowly, while that of mode A varies sharply, and the frequency split rises on the whole. It is noteworthy that the sensitivity of forming grooves on the top edge of the resonator wall is higher than that from drilling holes. For example, the frequency split of a resonator with grooves is 21.4 Hz when the trimmed mass is 1.053 mg, while split under drilling holes is just 4.5 Hz.

#### 3.3.2. Rigidity Condition

**Figure 8:**
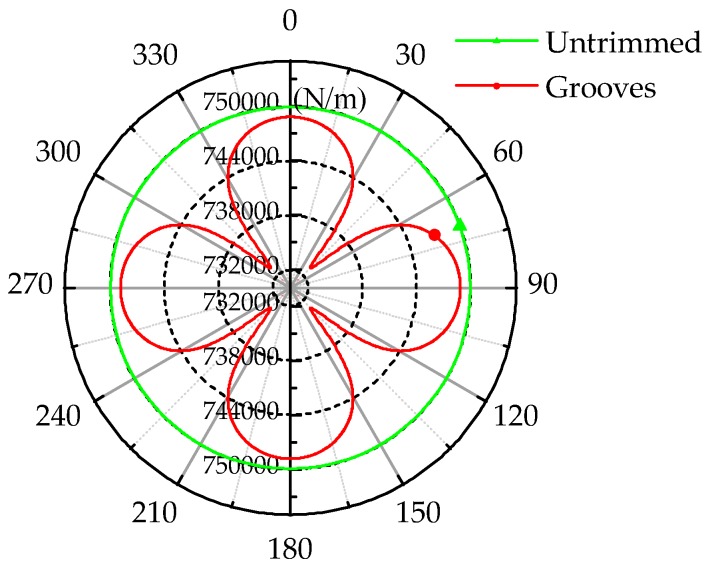
Rigidity condition of the resonator.

After stating the variation of eigenfrequency under grooves-trimming, it is necessary to research the rigidity variation under the same case. Equivalent radial rigidity is force over radial deformation, and deformation is produced by exerting force along the diameter of the wall of the resonator. The radial rigidity condition of the resonator (with a depth of grooves of 1 mm) is illustrated in Figure 8, which is the result of our simulation, and it displays the rigidity of untrimmed and grooves-trimmed resonators.

The radial rigidity of the untrimmed resonator is a full circle, as there are no defects in a perfect resonator and it thus has the same condition everywhere. In contrast, the radial rigidity of the grooves-trimmed resonator changes regularly. In the trimmed mode located at 45, 135, 225, and 315° separately, corresponding to Figure 8, the rigidity of the resonator falls after grooves-trimming, and the four trimmed positions result in a decrease larger than that in other positions. Compared with rigidity variation, mass variation affects eigenfrequency little. That is why the eigenfrequencies of trimmed and untrimmed modes decrease simultaneously and the eigenfrequency of the trimmed mode decreases more than that of the untrimmed mode under grooves-trimming.

### 3.4. Comparison and Trimming Process

#### 3.4.1. Comparison of Split under Different Trimming Methods

**Figure 9:**
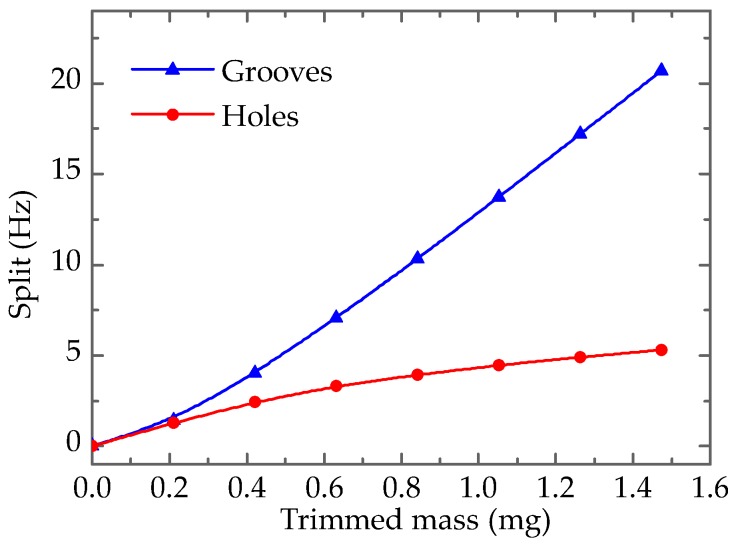
Frequency split of different trimming methods.

Figure 9 shows the frequency split under the two trimming methods (the areas of the trimmed outline are equivalent), which including drilling holes on the top and forming grooves on the top edge of the resonator wall. It is clear that grooving has a sharper effect on eigenfrequency, and drilling holes has a secondary sensitivity to eigenfrequency.

The sensitivity to eigenfrequency can be the main factor in choosing primary and precision trimming methods. In other words, when the frequency split is large, grooves-trimming can be employed to decrease it, and then holes-trimming can be utilized to eliminate the small residual split. In this way, the frequency split can be reduced to an appropriate level or be eliminated completely.

#### 3.4.2. Trimming Process

The simulation mentioned above involves trimming a perfect resonator to discover the variation principle of the resonator’s eigenfrequency. In order to validate the effect rules, a trimming process simulation for imperfect resonator was conducted; Table 3 presents the process of trimming.

A model of an imperfect resonator is built in FEM software to implement the trimming process, and its frequency split is 6.330 Hz. According to the effect rules of the different trimming methods, grooves-trimming is employed first in order to reduce the split, and then holes-trimming is utilized as a precision trimming method to eliminate the residual split. Grooves-trimming and holes-trimming are processed on mode A and mode B separately.

**Figure 10:**
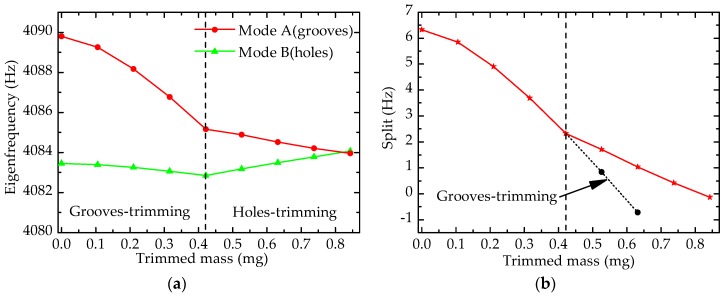
Simulation results of trimming process: (**a**) Eigenfrequency; (**b**) Frequency split.

As shown in Figure 10, the changing trends of eigenfrequency and frequency split are presented, and the curve on the left of the dashed line is the trend of grooves-trimming while the right is the trend of holes-trimming. On the left in Figure 10a, eigenfrequencies of both mode A and mode B decrease, and the eigenfrequency of mode A decreases significantly, which coincides with the effect rules of grooves-trimming. After trimming 0.421 mg by the grooves-trimming method, the frequency split deceases from 6.330 Hz to 2.319 Hz (decrease by 4.011 Hz). As shown in Figure 10b, the change trend on the left is more and more sharp, indicating that the efficiency of grooves-trimming increases gradually. In this case, trimming equipment with high-precision is demanded in practice to realize precision trimming, which would increase the cost of fabrication. Therefore, holes-trimming is employed.

According to Figure 10, the changing trend of holes-trimming is also in good agreement with the effect rules mentioned above. The sensitivity of holes-trimming is smaller than that of grooves-trimming, as shown in Figure 10b clearly. Holes-trimming is processed on mode B after grooves-trimming, and it only reduces the split by 1.838 Hz when the trimmed mass is also 0.421 mg (from 0.421 mg to 0.842 mg). In this way, the frequency split can be eliminated rapidly and precisely.

## 4. Experiments

In order to validate the simulation results, trimming experiments are implemented to verify the change rules of eigenfrequency. Two experiments are presented; one involves drilling holes on the top of the resonator wall, and the other involves forming grooves on the top edge of the wall.

### 4.1. Experimental Equipment

**Figure 11:**
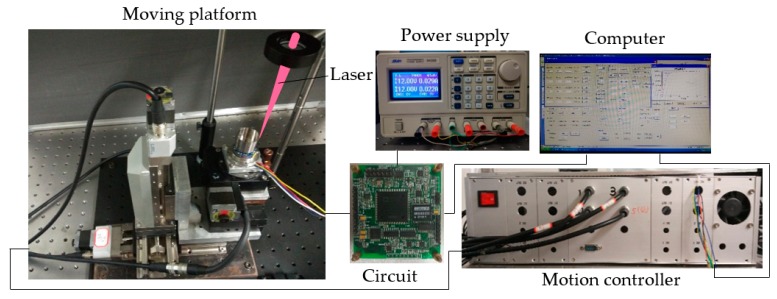
Experimental system.

A trimming platform system is set up to carry out the experiments, as shown in Figure 11. Traditional mechanical approaches such as drilling and milling are improper for thin cylindrical walls, hence the method of femtosecond laser ablation is employed to trim a tiny portion of material from the resonator. The laser source is a Ti:sapphire laser regenerative amplifier system (Spectra Physics Inc., Santa Clara, CA, USA), which provides a fundamental Gaussian mode with a central wavelength of 800 nm, pulse duration of 120 fs, and a repetition rate of 1 KHz. A power supply is connected to a controlling circuit board, which drives the resonator vibrating in the driving mode. A computer is utilized to analyze the signal via the circuit board from the gyroscope and govern the movement platform. The gyroscope is located on a movement platform according to the signal from the controlling circuit board, and the signal consists of the frequency and amplitude of the vibrating gyroscope.

### 4.2. Effect of Holes on Eigenfrequency

**Figure 12:**
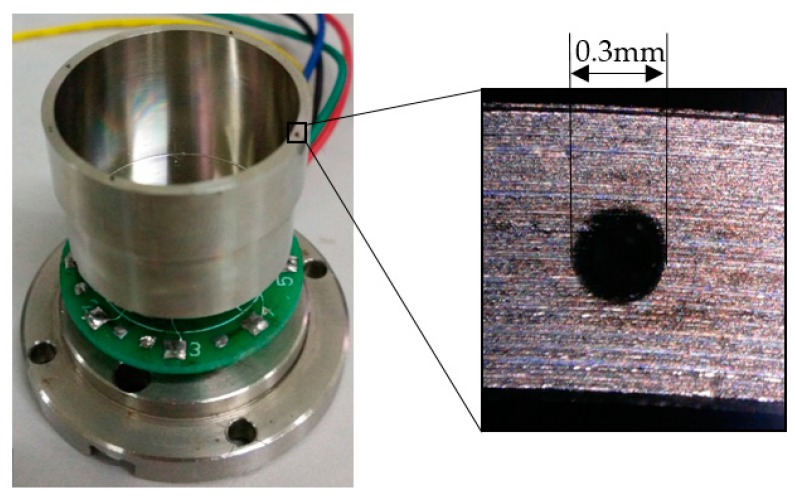
Drilling holes on the top of the wall in the experiment.

**Figure 13:**
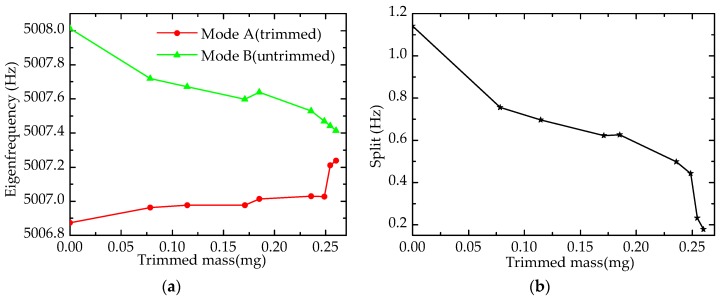
Experimental results of drilling holes on the top of the wall: (**a**) Eigenfrequency; (**b**) Frequency split.

Figure 12 describes the ablated outline of holes (Figure 13 shows the results), depending on the laser facula area. It is common to modify the ablation times in laser processing in order to control the trimmed mass, and exact mass can be calculated by the cross-section, depth, and shape of the hole.

At first, the eigenfrequency of the resonator is 5006.874 Hz (mode A) and 5008.014 Hz (mode B), respectively, and the frequency split is 1.140 Hz. With four holes ablated by the laser on the lower-frequency axis (mode A), the eigenfrequency of mode A increases gradually, and that of mode B decreases, which is similar to the simulation results. In the case of quantity, there is a certain error between the experimental results and simulation values. The reason for this is that model in the simulation is completely accurate, and materials are smooth and without defect, whereas the model in experiment has fabrication errors.

### 4.3. Effect of Grooves on Eigenfrequency

**Figure 14:**
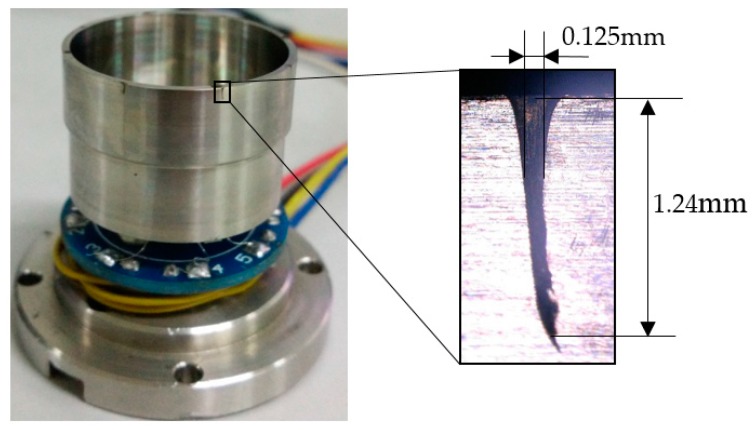
Forming grooves on the top edge of the wall in the experiment.

**Figure 15:**
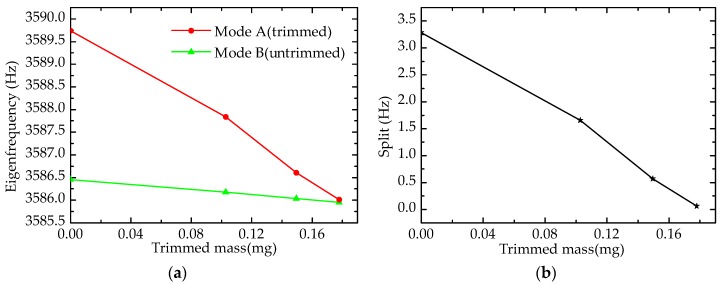
Experimental results of forming grooves on the top edge of the wall: (**a**) Eigenfrequency; (**b**) Frequency split.

As shown in Figure 14, four grooves are ablated on the top edge of the resonator, the results of which are presented in Figure 15.

Figure 15 describes the trends of eigenfrequency and frequency split; the eigenfrequency of mode A (trimmed) falls rapidly, whereas that of mode B decreases slowly, which leads to the elimination of the frequency split (0.06 Hz). This means the effect of grooves-trimming is also validated by our experiments. In addition, the experimental results proved that the sensitivity of grooves-trimming is higher than of holes-trimming by comparing the amount of decrease in the frequency split under same trimmed mass.

## 5. Conclusions

In summary, this paper presented the effects of different resonator-top trimming methods on eigenfrequency. The trimming methods are both processed on the top of the resonator wall; one is the holes-trimming method, and the other is the grooves-trimming method. First, a precise finite element model is established to obtain the numerical solution of the effects. According to the sensitivity of effect, a precision trimming method is introduced. After that, experiments are implemented to validate the numerical solution. It is noteworthy that a femtosecond laser is employed as a tool to trim the resonator in our experiments.

More specifically, the effects of different trimming methods on eigenfrequency are as follows. The holes-trimming method increases the eigenfrequency of the trimmed mode almost linearly, and decreases the eigenfrequency of the untrimmed mode slightly. The grooves-trimming method decreases the eigenfrequency of the trimmed and untrimmed modes simultaneously; nevertheless, the eigenfrequency of the trimmed mode varies more than that of the untrimmed mode, which leads to a further reduced frequency split when the eigenfrequency of the trimmed mode is higher. Besides, as the sensitivity to eigenfrequency of different trimming methods are sequenced as grooves-trimming and then holes-trimming, grooves-trimming can be a primary trimming method, and holes-trimming can be a precision trimming method. These simulation results are in good agreement with the experimental results, and the rigidity condition of the grooves-trimming coincides with the changes of eigenfrequency. In addition, the femtosecond laser exhibits excellent advantages in precise material processing.

In brief, frequency split can be eliminated by holes-trimming at a lower eigenfrequency mode and grooves-trimming at a higher eigenfrequency mode, and a femtosecond laser can be an efficient tool in resonator trimming. It is believed that these results will play a significant role in resonator trimming to improve the performance of cylindrical shell gyroscopes.

## Figures and Tables

**Table 1:** Material properties of the resonator.

Parameter	Value
Young’s modulus E	210 GPa
Poisson’s ratios μ	0.3
Density ρ	7800 kg/m^3^

**Table 2:** Geometry parameters of the resonator.

Parameter	Value (mm)
Height and thickness of resonant ring H1 and T1	8 and 1
Height and thickness of suspension ring H2 and T2	10 and 0.3
External radius of resonator R	13.5
Thickness of bottom H3	0.3
Internal, external, and height of substrate r1, r2, and H4	2, 4 and 4

**Table 3:** Trimming process in our simulation.

Trimming Method	Trimmed Mass (mg)	Eigenfrequency (Hz)		Split (Hz)
		Mode A (Grooves)	Mode B (Holes)	
Grooves-trimming	0	4089.791	4083.461	6.330
	0.105	4089.245	4083.399	5.846
	0.211	4088.159	4083.260	4.899
	0.316	4086.766	4083.074	3.692
	0.421	4085.169	4082.850	2.319
Holes-trimming	0.526	4084.885	4083.177	1.708
	0.632	4084.516	4083.489	1.027
	0.737	4084.201	4083.789	0.412
	0.842	4083.950	4084.080	−0.130
